# Indents

**Published:** 1921-06

**Authors:** 


### INDENTS

**☞Brief Articles of Technical or Scientific Value will receive notice and comment. Contributions invited.**

---

To Freshen Gold for Casting. Never use the same gold for casting more than once without cleaning. This can readily be done by making a metal casing for a carbon block to overcome block cracking under heat. Place gold in carbon block and while in molten condition add a mixture of one part of borax to three parts of potassium nitrate. This kept up for a few minutes will render gold in splendid condition for casting and good results can be expected.—W. D. N. Moore, *Dental Facts.*

---

Dangers in the Use of Adrenalin. The dentist in using this drug has the following object in view: to cause local anemia, *i. e.,* shutting off the blood supply to the field of operation. By so doing, two objects are gained. First, the anesthetic is confined locally and is not distributed over a wide area, so gaining a better anesthesia; second, the anesthetic is prevented from too quickly entering the blood stream.

*Dose.* This varies considerably. Prinz gives the dose 1 to 20,000. The most experienced operators are now advising against the injections of solutions containing more than one part of adrenalin to 40,000, and some even advise as low a dilution as one part in 100,000 or 150,000.

*Dangers.* (1) In the unstable nature of this drug: It is destroyed by light, therefore has to be stored in bottles which must be free from alkalies and acids. If these precautions are not taken, the drug must then be reduced to some unknown quantity, and the results may or may not be harmful. (2) Sloughing: This will occur more readily if this drug is used in conjunction with an anesthetic. By shutting off the blood supply to the parts the resistance of the tissues is lowered, and if the supply to the deeper structures is cut off necrosis of the hard tissues may even follow. (3) Secondary hemorrhage occurs, this fact has been pointed out by Prinz and other well known authorities. (4) Blood pressure: From time to time patients present themselves who suffer from high blood pressure, due to organic diseases. By these injections things are not made any more pleasant for these patients; in fact, their stay on earth is made rather uncertain. (5) Toxemia: When this drug is taken up by the blood stream it is sometimes followed by signs of toxemia. In many cases the symptoms shown are nausea, profuse sweating, fainting, muscular tremors and muscular contraction.

To-day the dose is reduced to almost nothing, and it is doubtful whether the amount is capable of producing the desired result, namely, local anemia. Many anesthetics are being placed upon the market to-day that contain adrenalin. Most of them state it plainly on the label, but do not state what percentage is used, nor when the solution was prepared.—R. A. Muir, D.D.S., *Commonwealth Dental Review.*

---

To Clean Gold Work after Polish. Hot water and Gold Dust soap and add one-half teaspoonful of ammonia for every pint of water.—Charles LeMyre, *Dental Facts.*

---

To Remove the Stain of Iodin. Iodin stain can easily be removed from dental napkins and other fabrics by washing the spots in ammonia water.—H. A. Cross, *Dental Facts.*

---

To Clean the Inside of Aluminum Plates White as Snow. Take about one teaspoonful of granulated lye, spread on the inside of the plate; leaving lye on one-half minute. Then wash (brush), with about a teaspoonful of hot water and Gold Dust soap.—Charles LeMyre, *Dental Facts.*

---

USE OF DENTAL FLOSS. Dental floss tied in two knots about one-half inch apart, and worked between the teeth, readily brings out all dirt, even to the largest pieces, which smooth floss will not do.—S. Adachi, *Dental Facts.*

---

"LET THERE BE LIGHT." While the dental profession is groping in the dark toward the solution of many difficult problems; while different methods of root-canal and pyorrhea treatments, bridge abutment, etc., have been suggested and tested with no satisfactory conclusion arrived at, there is a group of dentists who long ago solved all these problems to their own satisfaction and to the good of their patients (?). The only wonder is that our so-called leaders of the profession have not paid sufficient attention to this group and have not given their methods the publicity due them. If the average dentist plugging along in his office trying to make both ends meet and at the same time keep his conscience clear, if he knew of the most modern methods as practiced by the group mentioned above, he would not only be able to put away something for a rainy day, but would cheer up and forget about nervous breakdowns.

Now, I am not trying to do this group the justice they have been deprived of. I shall leave this to men to whom the English language is a more faithful servant than to me. All I wish is to call attention to the fact that a very great number of people, mostly poor, are being benefited (?) by this group of dentists, and that it is only fair that the less fortunate middle class and rich, who, by the way, pay bigger fees, should also enjoy these benefits. I really can not understand why in our magazines not a word is said about the so-called advertising offices or "Dental Parlors."

I wish to cite only a few of their rules and let the reader judge for himself:

1. Do not make any other kind of abutments except shell crowns.

2. The tooth does not necessarily have to be ground down. All you have to do is to separate the teeth.

3. Do not use "lightning disks" or safe-side disks to do the separation. Disks cutting on both sides are good enough.

4. If the band reaches too far under gum margin or does not reach it at all, you should worry.

5. If the bridge touches the opposite teeth only at certain points, that is fair enough occlusion.

6. If the abutment teeth are loose and pyorrhetic, make a bridge; it will stay for a while.

7. If the patient wants a crown on a loose and pyorrhetic tooth, make it. If you don't, somebody else will.

8. If a patient has one bicuspid on each side of the lower jaw, make a full bridge and guarantee it for twenty years. If later the bridge, together with the teeth, have to be removed, make him a plate free of charge and the patient will think you are a great sport.

9. Same with the upper.

10. Thirty-two gauge of some karat or other is good enough for crowns.

11. Never use solder higher than 18-k. of the cheaper grades.

12. Do not use inlays and foils except when the patient can not be talked out of them—use amalgams.

13. Make crowns in every case of a two-surface cavity.

14. If you must use amalgams, put in one filling in two adjoining proximal cavities—splendid contact.

15. Do not use any form of matrix for filling proximal cavities with amalgam—not necessary.

16. Never polish amalgams—waste of time.

17. Sterilize your instruments once a day. That is enough, if not too much.

18. Do not spend your hard-earned cash on saliva ejectors and cotton rolls. You have no use for them.

19. All the medicines you need in your office are: Iodin, oil of cloves, and a good-looking mouthwash supplied gratis by a salesman. If you treat teeth, have also arsenic and mummifying paste or pustolene.

20. Root-canal technique is as follows (if you are not a hundred "percenter"):

1st Sitting: Put in arsenic and seal with gutta-percha stopping.

2d Sitting: Open into canals, extirpate as much of the pulp as possible, let patient spit; somehow or other dry the canals and pump in mummifying paste. Put crown on. Very simple.

(By the way, the man who advocates this technique on having been told about focal infection, etc., said that if you treat a tooth right, you have nothing to fear, and that it is only the young, inexperienced dentists who make such a fuss over nothing.)

21. In case of pyorrhea, extract all teeth that you do not need for abutments.

22. Prepare your own local anesthetic, half a pint or more at a time. If flakes form in a few days, pass it through a cloth, if you are particular.

23. If you wish to sterilize your forceps before extraction, just moisten the tips of the beaks with iodin and you are safe. The same with the needle if you wish to be so careful.

24. If you extract teeth for a bridge, do not wait for the gum to heal. While the patient is bleading, make the abutments, take bite and impression and make the bridge. Or make abutments, take bite and impression and then extract the teeth.

25. Buy an X-ray machine and let the cashier "take the pictures" and diagnose the cases. Shortened or elongated, distinct or not, the teeth or parts of them will be on the picture and if there is a dark area anywhere, the cashier knows that it is an abscess and the tooth has to be pulled. If she happens to hit on the antrum, the operator may sometimes correct her. The X-ray is a good advertisement.

These are only a few of the suggestions that save time, money and worry and are of great benefit to the patient. Will any of our leaders consider them and so to speak revolutionize dentistry?

There are also excellent and legitimate means of advertising, but of these later.—*Cosmos.*

---

Ethical Parables. The philosopher was compelled to wait in one office until the close of the business day, but he was so impressed by the atmosphere of comfort and quiet efficiency, and took such pleasure in watching what he could see of the office workings that he found this no hardship. When the dentist was free, he explained that he was seeking an application of ethics to the practice of dentistry.

"I can not pretend to give you that," said the dentist, "but I can tell you one or two things that I have learned by sad experience.

"The most important thing I have learned is to see no more people than I can give undivided attention. I used to see fifteen or twenty people a day. I hurried for each one and consequently formed many wrong or incomplete decisions and performed many operations less well than I might have done by taking more time.

"I saw the folly of this from all points of view, and gradually turned over a new leaf. Now I give to each person undivided attention, which can be broken in on only by emergency cases. No thoughts of my own affairs are allowed to intrude, because I have arranged them so that they need not.

"I take time enough for each case, all the time it needs. If a patient desires an advance estimate, I include enough time so that I can make all the models and do all the study I wish. I know that every moment is paid for.

"I have far fewer patients than in the old days, but all seem satisfied. And I am happy because I know that very few pieces of work leave the office which are not the best I can do."—George Wood Clapp in *Digest*.

---

PROPHYLACTIC AID. Phenol sodique as aid in preventing fermentation of food debris and healing injured parts is frequently lost sight of. As a local application for pus pockets in pyorrhea alveolaris, it is hard to beat. It can always be relied upon as a very effective styptic and anodyne after extraction.—F. W. Frahm, *Pacific Gazette*.

---

SULPHURIC ACID AND SODIUM POTASSIUM IN ROOT-CANAL CLEANING. A forty or fifty per cent. solution of sulphuric acid in water will give the desired effect in removing or breaking down the inorganic part of the canal wall. Using two or three drops of the acid at the entrance to the canal and then pumping it in with a smooth broach. At the next sitting alternate this treatment with the sodium potassium paste for the purpose of removing or disintegrating the organic substance of the canal wall. Continue these alternate applications until the canal is opened sufficiently for a good filling.—F. W. Frahm, *Pacific Gazette*.

---

THE EFFECTIVENESS OF TOOTH WASHES. There are few persons now who have not the conviction that the scientific cleansing of the teeth and gums is very valuable, and the business in antiseptic dentifrices is obviously increasing, judging from the numerous public advertisements which appear in the daily press setting forth the merits of a great number of these preparations. Generally speaking, they are so composed as to render them well adapted for the purpose, and we have submitted a number of them to analytical examination. The choice of antiseptic is commonly confined to carbolic acid, thymol, peppermint, boric acid, benzoic acid, eucalyptus and other essential oils, distributed in vehicles containing as an agent of attrition nongritty chalk and as an alkaline medium soap. Experiments are usually quoted as to the actual germicidal value of these preparations *in vitro,* but, of course, it would be more satisfactory to have evidence of their real effect in the mouth, where very different conditions must, as a matter of fact, exist. The use of a tooth wash does not approach the conditions of a laboratory test, though there can be little doubt that a good deal of germicidal work in the mouth is done by the vigorous application of the toothbrush, and it may be pointed out that the tongue may well be included in the process. To be effective, however, the action of all antiseptics takes time, according to the vitality of the organisms they encounter, and usually the toothbrushing process does not occupy many seconds. The question of time-exposure is important, but it is very generally overlooked, and consequently the antiseptic treatment of the teeth falls short of that effectiveness which is shown to be the case in laboratory experiments. The toothwashing process should be more prolonged, and the antiseptic wash allowed to remain in contact with the teeth and gums for some minutes, instead of seconds, before finally washing the mouth clear of antiseptic with plain water.—Lancet, *Oral Health.*

---

SYNTHETIC FILLINGS. Where one has to do a synthetic or oxyphosphate filling and where it is impossible to use the rubber dam, paint the surrounding gum with dilute trichloracetic acid; this will stop the exudation of moisture from the gum, and work can then be carried out in a dry area without fear of having the work spoiled by moisture. —G. Levin, *Ash's Dental Magazine.*

---

To Light Gas without a Match. Some time you may want to light your gas or alcohol lamp quickly and have no match within reach of your free hand. If your electric cautery is convenient it will do the lighting for you.—J. F. Nelson, *Dental Facts.*

---

To Prevent Checking of Porcelain in Casting. When casting on porcelain turn the investment over immediately, so the button of surplus gold is excluded from the air, allowing the whole to cool evenly. The facing will not check when this is done promptly. This also applies to solder work.—J. S. Bridges, *Dental Facts.*

---

Ethanesal. As the result of prolonged and laborious experimentation, a new anesthetic has been produced by Dr. R. L. Mackenzie Wallis from highly-purified ether, and with the addition of certain other substances. A full account of the investigation and the results attained was given in a paper read at a meeting, held on March 5th, of the Royal Society of Medicine, in the Section of Anesthetics. In the various stages of development of the research not the least difficult was the preparation of a pure ether, which, however, possesses no anesthetic properties. After a long and concentrated search, it was found that the anesthetic action was due to the higher ketones, which also retained in the ether the carbon dioxide and ethylene gases that other investigators had found to be necessary additions. The carbon dioxide and ethylene were introduced, along with the ketones, into the pure ether. The practically odorless anesthetic made in this way by Dr. Mackenzie Wallis was tried with specially successful results on a large number of cases by Mr. Langton Hewer. It was since found impossible to cope with the demand for this new anesthetic, and so recently its manufacture has been taken up by a well-known chemical firm, and to their preparation they have given the name of "ethanesal."—*Dental Record.*

---

THE OFFICE COAT. I think it is about time to discard the nasty, long-sleeve jackets and replace them with surgeons' operating gowns in every office. Just imagine thrusting the same sleeve into, say, a dozen patients' faces! —*Oral Hygiene.*

---

DEATH FROM APLASTIC ANEMIA. The untimely and widely regretted death of Dr. William Ironside Bruce, who succumbed from aplastic anemia after a prostrating illness brought on by self-sacrificing devotion to radiographic work, has brought home to the minds of many of us the real gravity of the dangers to which X-ray workers are exposed. His loss will be specially felt by many friends and the staff at Charing Cross Hospital, where he had worked and taught so well, and where his long experience, knowledge and keenness had been mainly responsible for bringing the X-ray department to a fine pitch of efficiency in diagnosis and treatment.

A good many years ago several investigators showed that the blood and normal tissues of animals exposed to X-rays underwent marked changes. It was found also that some disturbance was produced even in the bone-marrow, and more recently it has been proved that the blood of an individual who has been exposed to fractional but repeated doses of the penetrating radiations suffers a serious loss in the red cells as well as the circulating leucocytes. The graver effects of such exposure to the more penetrating type of rays may culminate, it is believed, in damage being done to the blood-forming organs. In view of the serious issues at stake, it is earnestly hoped that radiologists will give an increasingly larger share of their attention to the difficult task of finding the best means of eliminating the risks of personal injury and of safeguarding the health of the operator.—*Dental Record.*

---

Dental Ideals. If you have determined to fit yourself to hold a worthy place, you need not be fearful of the future. Friends will form around you to aid to your advancement, and sooner or later you will be "greeted with grateful whispers" and voices of praise for your professional successes.

"Be not satisfied to rest upon your present laurels, lest they wither while you yet caress them." Let few hours be wasted in indolent ease; be earnest searchers after truth. Always have on hand something that you are investigating; such employment of your spare moments will add freshness and interest to your life, it will keep the intellect active, and benefit not only yourselves but those with whom you associate.—J. N. Broomell, in *Oral Hygiene.*

---

Intellectual Securities. Wise and fortunate indeed is the student of dentistry who regards his professional course as an investment in intellectual securities. Every scrap of knowledge he stores away, every art of technique he masters, every high ideal of professional life he develops becomes the invested capital upon which his future success depends. Its value is derived almost wholly from the extent and character of the efforts he has put forth.

Happily this intellectual treasure once acquired may not be lost through adversity, nor is it diminished when shared with others. It is forever safe and productive, and one of the few human possessions which neither moths nor rust can corrupt nor thieves break through and steal.—Dr. Charles K. Turner, in *Oral Hygiene.*

Bachelor of Dental Surgery. It is reported that London University is to institute a new degree in dentistry. The M.D.S. (Lond.) degree was established in 1909, but so far not a single candidate has presented for examination. London University has now decided to fall into line with the Universities of Birmingham, Bristol, Dublin, Ireland (National), Liverpool and Manchester, and to establish, in addition to the Mastership, a Bachelorship of Dental Surgery, for which the previous acquisition of a degree in general medicine or surgery will not be obligatory. It may confidently be expected that at least a fair proportion of men will take the necessary steps to secure the B.D.S. (Lond.).—*Dental Record.*

---

The Dual Nature of Apothesine. In a "study of the antiseptic action of certain local anesthetics," Macht, Satani and Swartz, of the Pharmacological Laboratory of Johns Hopkins University and the James Buchanan Brady Iustitute, of Baltimore (*The Journal of Urology,* August, 1920), announce some very important facts having a significant bearing upon the use of these agents in cases in which infections exist or may supervene. The drugs subjected to the tests were cocaine, novocaine, stovaine, alypin, holocaine, alpha-eucaine, beta-eucaine, apothesine, and benzyl alcohol. They were dissolved in physiologic salt solution in various concentrations, and their antiseptic action was observed first on the Staphylococcus aureus and B. coli, and afterwards, with the exception of cocaine and novocaine, on the gonococcus. The authors say "it is interesting to note that our two best known and most widely used local anesthetics, namely cocaine and novocaine, are entirely devoid of any antiseptic action." Because of this fact these two drugs were not tested against the gonococcus.

It was found, however, that "Apothesine killed the gonococcus in five minutes in strengths of 1, 1.5, and 2 per cent., or over.'' In solutions as weak as one-half of one per cent. but a few colonies survived, and ''these did not appear for forty-eight hours, showing an inhibitory or antiseptic action even in 0.5 per cent. solution.''

From the work of these investigators it is evident that Apothesine is not only a valuable anesthetic but a dependable antiseptic and germicide as well. It would seem, therefore, that it should be the anesthetic of choice in all operations in which the use of a general anesthetic is inexpedient and the prompt healing of the wound, without suppuration, the desideratum. The possession of definite antiseptic value in the anesthetic would do away with the necessity of a preliminary sterilization of the solutions. It might be added that, because of the antiseptic action of the substance, stock solutions of Apothesine keep well.—P. D. & Co., *Notes*.

---

ORAL HYGIENE FOR STUDENTS. If every student who is graduated from a dental college in America had a just conception of the value of oral hygiene, and was sufficiently impressed with its importance to carry it out in his daily work when he enters practice, the aggregate result in a few years would be a lessened tendency to dental disease, and an increase of efficiency among the people served by these graduates. But the greatest good in oral hygiene can never come from the services of the dentist alone. Not till the public generally are sufficiently educated to recognize the benefits of oral hygiene, and are willing to cooperate with the dentist and do their share day after day in the way of oral cleanliness, can the best results be obtained. It is therefore as much a matter of education as it is one of technical procedure, and the dentist who is doing the greatest good is the one who, in addition to the services he renders at the chair, is daily inculcating into his patients the ideas of prevention as embodied in oral hygiene.—C. N. Johnson, in *Oral Hygiene*.

